# Case Report: Alopecia areata in an adolescent after COVID-19 infection

**DOI:** 10.3389/fped.2026.1723836

**Published:** 2026-03-26

**Authors:** Han Xu, Yunhui Pan, Yinfang Tu, Haoyong Yu

**Affiliations:** 1 Department of Endocrinology and Metabolism, Shanghai Sixth People’s Hospital Affiliated to Shanghai Jiao Tong University School of Medicine, Shanghai Diabetes Institute, Shanghai Key Laboratory of Diabetes Mellitus, Shanghai Clinical Center for Diabetes, Shanghai, China; 2 Community Health Service Center of Caohejing Street, Shanghai, China

**Keywords:** adolescent, alopecia areata (AA), COVID-19, glucocorticoids, infection, minoxidil

## Abstract

COVID-19 infection may trigger the onset or progression of various autoimmune disorders, although the exact pathogenesis remains poorly understood. We report the case of a girl presenting with patchy hair loss 1 week after a confirmed COVID-19 infection, who was subsequently diagnosed with alopecia areata (AA). Serum autoantibody testing returned negative results. Treatment involved external application of topical glucocorticoids and minoxidil to stimulate hair regrowth. After 6 months of therapy, a significant improvement in hair density was observed across the scalp, with only a single recalcitrant patch of AA remaining. Given the increasing prevalence of COVID-19, the potential for viral-induced AA warrants clinical attention.

## Background

Since its emergence as a global pandemic more than 6 years ago, COVID-19 has been associated with a wide range of postinfectious sequelae. The WHO-led Delphi Consensus defines post-COVID-19 condition as a syndrome typically occurring 3 months after the initial illness, with symptoms persisting for at least 2 months that cannot be explained by an alternative diagnosis. This condition is characterized by fatigue, dyspnea, cognitive impairment, and broader autoimmune dysfunction. These symptoms may emerge following initial recovery, persist from the acute phase, or fluctuate in severity over time, significantly impacting daily life.

Emerging evidence implies that COVID-19 may precipitate or exacerbate various autoimmune skin diseases. The proposed mechanisms include molecular mimicry, dysregulated cytokine signaling, and heightened Th1/Th17 immune responses, which collectively contribute to a loss of self-tolerance and the subsequent production of autoantibodies (1). While several cases of alopecia areata (AA) triggered by COVID-19 have been documented, the majority of reports have involved adult patients; the occurrence of AA in pediatric or adolescent populations remains a rare clinical phenomenon.

## Case presentation

The patient, an early adolescent girl, presented with progressive hair loss for more than 3 months. Her symptoms began with a fever of up to 39°C and a cough; she was diagnosed with COVID-19 based on a nasopharyngeal swab test at a local hospital. She did not have any obvious sputum, dyspnea, nausea, or vomiting. The fever subsided to normal after she took antipyretic medication.

One week later, she experienced sudden hair loss, which initially measured 1 cm × 1 cm. This gradually progressed to patchy, non-scarring alopecia in round and oval areas with clear boundaries. The edges of the hair loss areas were loose and her hair could be pulled out easily; the patient also reported occasional itching. The affected skin had no scales or peeling. There was no visible loss of eyebrows, eyelashes, or other body hair. The girl's fingernails had pinpoint depressions on the surface, although no photographs were taken at the time. In addition, she felt very anxious and distressed. There was no family history of similar conditions.

**Figure 1:**
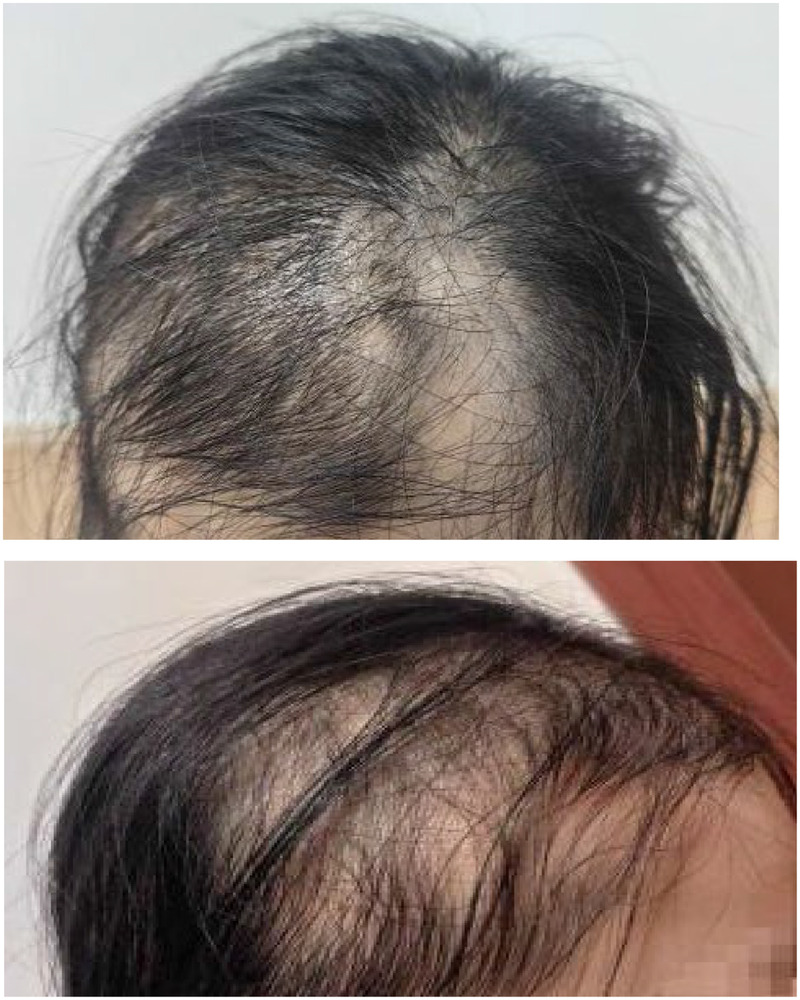
Frontal and side views of the patient with alopecia areata after COVID-19.

The patient was diagnosed with AA at a dermatology department based on the appearance of the hair loss. She was prescribed minoxidil tincture and halometasone ointment to be applied twice a day externally, along with the Janus kinase (JAK) inhibitor, ritlecitinib. A physical examination showed thinning hair and scattered round and oval alopecia areas 3–5 cm in diameter. The hair-pulling test was positive, and there were no white scales or redness (Figure 1). This presentation of focal patches and nail depressions is different from telogen effluvium (TE). The results of auxiliary tests were as follows: anti-single-stranded DNA (quantitative) 5.42 IU/mL, anti-double-stranded DNA antibody <10.0 IU/mL, anti-nuclear antibody (titer) <1:100, anti-U1-RNP/SM (−), anti-SM (−), anti-SS-A (−), anti-Ro-52 (−), anti-SS-B (−), anti-Scl-70 (−), anti-PM-Scl (−), anti-JM-Scl (−), anti-Jo-1 (−), anti-milus (−), anti-PNCA (−), anti-nucleosome (−), anti-histone (−), anti-ribosomal P protein (−), anti-double-stranded DNA antibody (−), and anti-mitochondrial antibody-M2 (−).

**Figure 2:**
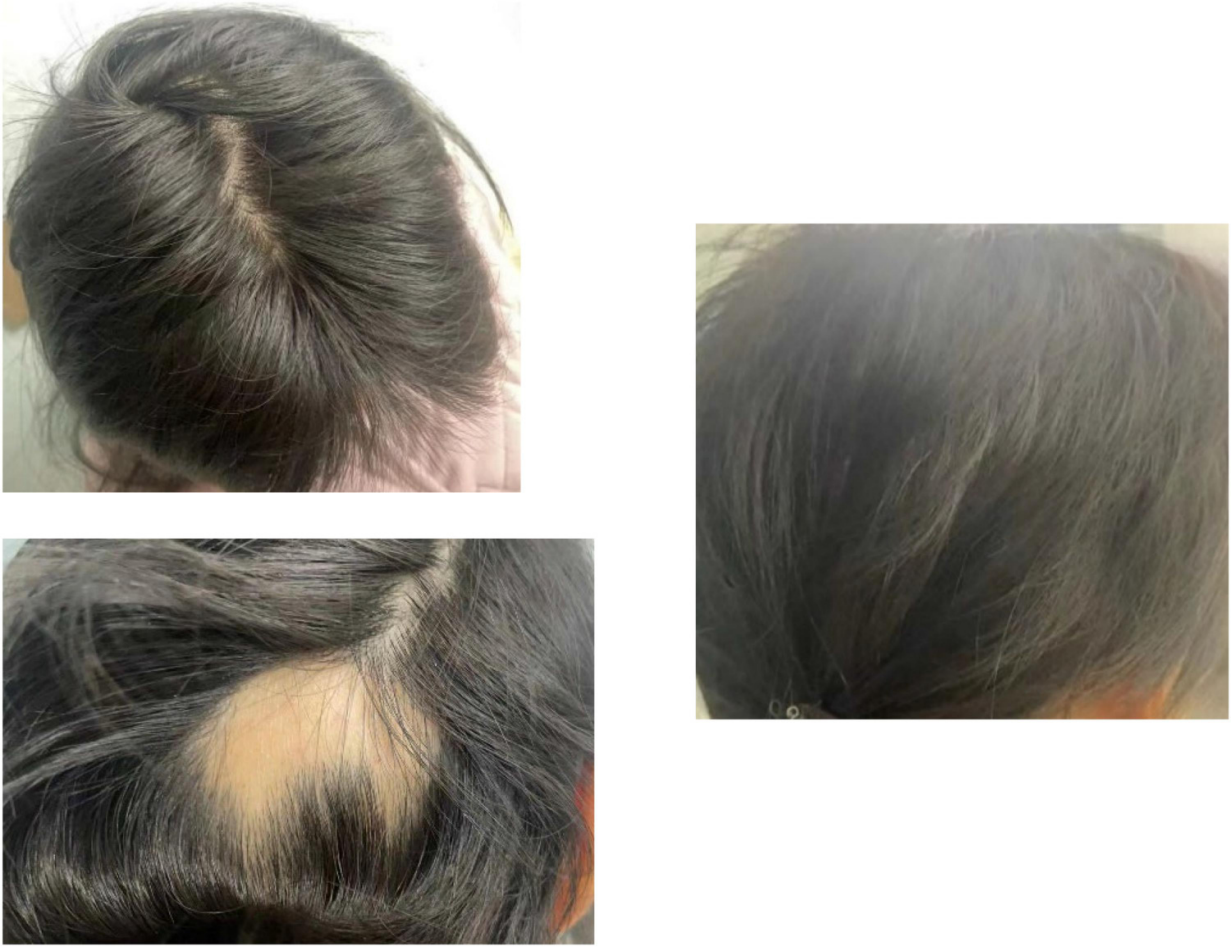
Frontal and side views of the patient with alopecia areata after 6 months of treatment.

After 6 months of treatment, total hair volume increased, with only one round area of AA remaining without hair growth (Figure 2). It has been 2 months since she stopped treatment, and there has been no recurrence of the disease.

## Discussion

The reported duration of post-COVID-19 syndrome symptoms in children varies across studies. A meta-analysis reported that the median duration of symptoms was 125 days after acute infection (P25 to P75: 99–231 days) (2, 3). A recent update from the UK Office for National Statistics (ONS) reported 117,000 children with post-COVID-19 syndrome, with 14,000 of those children experiencing symptoms lasting longer than 12 months (4). Differences in duration may be linked to varying pathogenetic mechanisms across clinical phenotypes. The most supported theories suggest that post-COVID-19 syndrome occurs as an autoimmune process, as the majority of patients exhibit significantly elevated serum levels of proinflammatory cytokines.

AA is a common T-cell-mediated autoimmune non-scarring alopecia, usually presenting as patchy hair loss. The pathophysiological basis is thought to be a disruption of immune regulation in hair follicles, leading to increased aggregation of natural killer cells. The majority of studies have confirmed genetic susceptibility for childhood patchy hair loss, which can also be induced by emotional stress, medications, viral infections (e.g., influenza, cytomegalovirus, or herpesvirus), and malnutrition, such as deficiencies in zinc, iron, and calcium. The diagnosis of AA is based on clinical examination and dermoscopy; typical features include the black spot sign, yellow spot sign, broken hairs, exclamation-mark hairs, and clusters of short hairs under magnification. According to the 2020 Alopecia Areata Consensus of Experts (ACE) study (5), the prognosis is poorer for patients with childhood onset, long disease duration, involvement of the temporal or perioccipital scalp, large areas of hair loss, recurrence, nail damage, concurrent autoimmune diseases, or a family history of AA.

Current treatments range from watchful waiting (as up to half of patients may spontaneously heal within 1 year) to active interventions. Common topical treatments include glucocorticoids, intralesional glucocorticoid injections, local immunotherapy, calcineurin inhibitors such as tacrolimus, and topical minoxidil to induce hair regrowth. Systemic treatments include oral glucocorticoids, immunosuppressants, and JAK inhibitors such as tofacitinib or baricitinib, as well as traditional Chinese medicine; however, caution should be taken with oral glucocorticoids in pediatric patients.

In this case, the patient had a clear trigger for disease onset: “progressive alopecia for more than 3 months” following a COVID-19 infection. The diagnosis of COVID-19-induced AA was established based on the characteristic round and oval areas of hair loss, a positive hair-pulling test, and negative autoantibody tests. According to the 2019 Chinese guidelines for the diagnosis and treatment of AA, topical glucocorticoids are the first-line therapy. For this severe case, ritlecitinib, a dual JAK3/TEC inhibitor, was taken orally, supplemented with topical minoxidil. The girl was also instructed to maintain a light diet, avoid emotional stress, and ensure sufficient sleep. While there are some reports of AA following COVID-19 infection or vaccination, these remain limited to case reports. Herzum et al. reported COVID-19-induced AA in pediatric patients and collected data on four children (6) (see Table 1), although the text did not provide details on efficacy and prognosis.

**Table 1:** Summary of cases of children presenting with alopecia areata after COVID-19 infection.

Source of information	Gender	Age (years)	Time to AA after coronavirus infection (weeks)	Size and location of AA
Herzum et al. (6)	Male	13	4	On the occipital bone; 1 cm × 1 cm
Herzum et al. (6)	Male	8	7	On the parietal lobe; 3 cm × 4 cm
Herzum et al. (6)	Male	9	4	On the top of the head; 3 cm × 3 cm
Herzum et al. (6)	Female	8	3	On the top of the head and along the marginal hairline; 2 cm × 2 cm

Based on the case reports available, the identified patients, including the girl in this case, were aged 8–13 years old. All patients developed AA following COVID-19 infection. While previous cases were relatively mild and controlled with topical glucocorticosteroids (notably in children with no prior personal or family history of autoimmune disease), the disease in the present case progressed more rapidly.

Although the exact pathophysiology remains unclear, the condition appears to be linked to an autoimmune response against the hair follicle. One hypothesis suggests that molecular mimicry may occur, potentially due to the capacity of a vaccine or virus to elicit production of the spike protein (7). Several studies have noted that AA is often associated with other autoimmune disorders, such as vitiligo, which supports an autoimmune etiology (6, 8). A comparative analysis found similar elevations of C-X-C motif chemokine ligands (CXCL9, CXCL10, and CXCL11), interferon gamma (IFN-*γ*), interleukin-10 (IL-10), tumor necrosis factor alpha (TNF-α), and interleukin-18 (IL-18) in patients with COVID-19 and other inflammatory conditions (9).

Viral infections such as COVID-19 may drive autoimmune pathogenesis through two primary mechanisms. First, they can induce oxidative stress, leading to the upregulation of major histocompatibility complex I (MHC-I) ligands on hair follicles. This triggers T-cell activation, follicular destruction, and the release of perifollicular IFN-γ and TNF-α. In genetically predisposed individuals, this can initiate self-reactive T-lymphocyte activity. Second, the COVID-19 virus may carry antigens that cross-react with host antigens, shifting the immune response to target the patient's own tissues.

Psychological stress following infection is another recognized risk factor for AA (8, 10, 11). As AA progresses, the visible loss of hair can significantly impact a patient's appearance, leading to low self-esteem and social withdrawal. Children are particularly vulnerable to these psychological changes. The 2019 Chinese guidelines for the diagnosis and treatment of AA emphasize that, along with medication, it is vital to manage mental stress, ensure adequate sleep, maintain a balanced diet, and engage in physical exercise. For patients who do not respond well to medication or lack a tendency for spontaneous healing, wigs or hairpieces may be used for concealment. While the majority of studies have confirmed genetic susceptibility for childhood patchy hair loss, triggers such as emotional stress, medications, viral infections, and micronutrient deficiencies (zinc, iron, and calcium) also play a role (12–14).

Our case report has some limitations. We cannot establish a definitive causal relationship; pediatric AA may have other triggers, and our case lacked dermoscopic images and detailed genetic and immunological profiling data. In conclusion, as COVID-19 and similar viral infections become more prevalent, new-onset AA warrants continued clinical attention.

## Data Availability

The original contributions presented in the study are included in the article/Supplementary Material, further inquiries can be directed to the corresponding author.
